# Supplementary material for: Environment-dependent epistasis increases phenotypic diversity in gene regulatory networks
Source: Sci Adv. 2023 May 24;9(21):eadf1773. doi: 10.1126/sciadv.adf1773 (PMC10208579; doi:10.1126/sciadv.adf1773)
Supplement: Supplementary file 1 — Figs. S1 to S14 Legend for source data [file sciadv.adf1773_sm.pdf]

Science Advances
AAAS

Supplementary Materials for

Environment-dependent epistasis increases phenotypic diversity in gene regulatory networks

Florian Baier *et al.*

Corresponding author: Joshua L. Payne, joshua.payne@env.ethz.ch; Yolanda Schaerli, yolanda.schaerli@unil.ch

Sci. Adv. **9**, eadf1773 (2023)
DOI: 10.1126/sciadv.adf1773

The PDF file includes:

Figs. S1 to S14
Legend for source data

Other Supplementary Material for this manuscript includes the following:

Source data

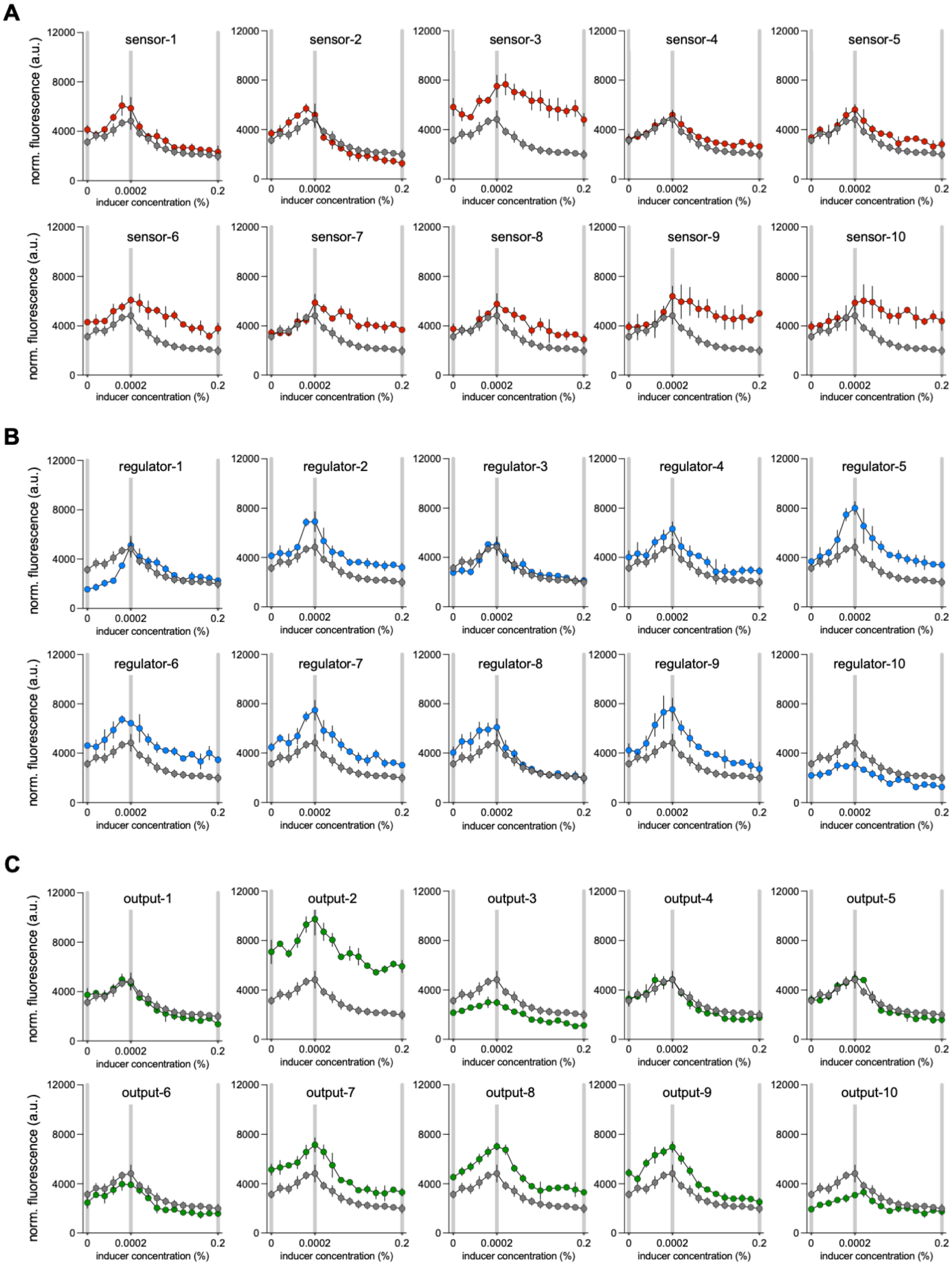

Fig. S1. GFP expression patterns of the selected 30 genotypes. They carry mutations in the (**A**) sensor, (**B**) regulator or (**C**) output node. GFP expression of the WT network is shown in grey. Each genotype was measured in triplicate at 16 inducer concentrations and the mean and standard deviation from three biological replicates are shown.

A

Genotype	AraC binding	-35	promoter pBAD	-10
WT	ATAGCATTTTTATCCATAAGATTAGCGGtTCCTACC	TGACGC	TTTTTATCGCAACTCTC	TACTGT
S1	ATAGCATTTTTATCCATAAGATTAGCGGTTCCTACC	TGACGT	TTTTTATCGCAACTCTC	TACTGT
S2	ATAGCATTTTTATCCATAAGATTAGCGGATCCTACC	TGACGC	TTTTTATCGCAACTCTC	TACTGT
S3	ATAGCATTTTTATCCATACGATTAGCGGTTCCAACC	TGACGC	TTTTTATCGCAACTCTC	TACTGT
S4	ATAGCATTTTTATCCATAAGATTAGCGGTTCCTACC	TGACGC	TTTATATCGCGACTCTC	TACTGT
S5	ATAGCATTTTTATCCATAAGATTAGCGGTTCCTACC	TGACGC	TATTTATCGCAACTCTC	TACTGT
S6	ATAGGATTTTTATCCATAAGATTAGCGGTTCCTACC	TGACGC	TTTTTATCGCAACTCTC	TACTGT
S7	ATAGCATTTTTATCCATAAGAGTAGCGGTTCCTACC	TGACGC	TTTTTATCGCAACTCTC	TACTGT
S8	ATAGCATTTTTATCCATCAGATTAGCGGTTCCCACC	TGACGC	TTTTTATCGCAACTCTC	TACTGT
S9	ATAGCATTTCTATCCATAAGATTAGCGGTTACTACC	TGACGC	TTTTTATCGCAACTCTC	TACTGT
S10	ATAGCATTTTTATCCATAAGATTAGCGGATCCTATC	TGACGC	TTTTTATCGCAACTCTC	TACTGT

B

Genotype	-35	promoter J23114	-10	spacer	tetO operator
WT	TTTATG	GCTAGCTCAGTCCTAGG	TACAAT	GCTAGCGAAGGG	TCCCTATCAGTGATAGAGA
R1	TTTATG	GCTAGCTCAGTCCTAGG	TACAAT	GCTAGCGAAGGG	TCCCTATCAGTGATAGAGT
R2	TTTATG	GCTAGCTCAGTCCTAGG	TACAAT	GCTAGCGAAGGG	TCCCTTTCAGTGATAGAGA
R3	TTTATG	GCTAGCTCAGTCCTGGG	TACAAT	GGTAGCGAAGGG	TCCCTATCAGTGATAGAGA
R4	TTTATG	GCTAGCTCAGTCCTAGG	TACAAT	GCTAGCGAAGGT	TCCCTGTCAGTGATAGAGA
R5	TTTATG	TCTAGCTCAGTCCTAGG	TACAAT	GCTAGCGAAGGG	TCCCTATCAGTGATAGAGA
R6	TTTATG	GCTAGCTCAGTCCTAGG	TACAAT	GCTAGCGAAGGG	TCCCTATCAGTGTTAGAGA
R7	TTTATG	GCTAGCTCAGTCCTAGG	TACAAT	GCTAGCGAAGGG	TCCCTATCAGTGATACAGA
R8	TTTATG	GCTAGCTAAGTCCTAGG	TACAAT	GCTAACGAAGGG	TCCCTATCAGTGATAGAGA
R9	TTTATG	GCTAGCTCAGTCCTAGG	TATAAT	GCTAGCGAAGGG	TCCCTATCAGTGATAGAGA
R10	TTTATG	GCTAGCTCAGTCCTAGG	TACAAT	GCTAGCGAAGGG	TCCCTATTAGTGATAGAGA

C

Genotype	-35	promoter J23100	-10	spacer	lac operator
WT	TTGACG	GCTAGCTCAGTCCTAGG	TACAGT	GCTAGCGAAG	GGAATTGTTATCCGGATAACAATTCC
O1	TTGACG	GCTAGCTCAGTCCTAGG	TAGAGT	GCTAGCGAAG	GGAATTGTTATCCGGATAACAATTCC
O2	TTGACG	GCTAGCTCAGTCCTAGA	TTCAGT	GCTAGCGAAG	GGAATTGTTATCCGGATAACAATTCC
O3	TTGACG	GATATCTTAGTCCTAGG	TACAGT	GCTAGCGAAG	GGAATTGTTATCCGGATAACAATTCC
O4	TTGACG	GCTAGCTCAGTCCTCGG	TACAGT	GCTAGCGAAG	GGAATTGTTATCCGGATAACAATTCC
O5	TTGCCG	GCTAGCTCAGTCCTAGG	TACAGT	GCTAGCGAAG	GGAATTGTTATCCGGATAACAATTCC
O6	TTGACG	GCTAGCTCAGTCCTAGG	TACAGT	GCTAGCGCAG	GGAATTGTTATCCGGATAACAATTCC
O7	TTGACG	GCTAGCTCAGTCCTAGG	TACAGT	GCTAGCGAAG	GGAATTGTTATCCGAATAACAATTCC
O8	TTGACG	GCTAGCTCAGTCCTAGG	TACAGT	GCTAGCGAAG	GGAATTGTTATCCGGATGACAATTAC
O9	TTGACG	GCTAGCTGAGTCCTAGG	TACAGT	GCTAGCGAAG	GGAATTGTTATCCGGATACCAATTCC
O10	TTGACG	GCTAGCTCAGTCCTAGG	TACAGT	GCTAACGAAG	GGAATTGTTATCCGGATAACAATTCC

.

Fig. S2. **Nucleotide changes in promoter and operator regions of the selected genotypes.** Mutations (in color) of the (**A**) sensor, (**B**) regulator or (**C**) output genotypes.

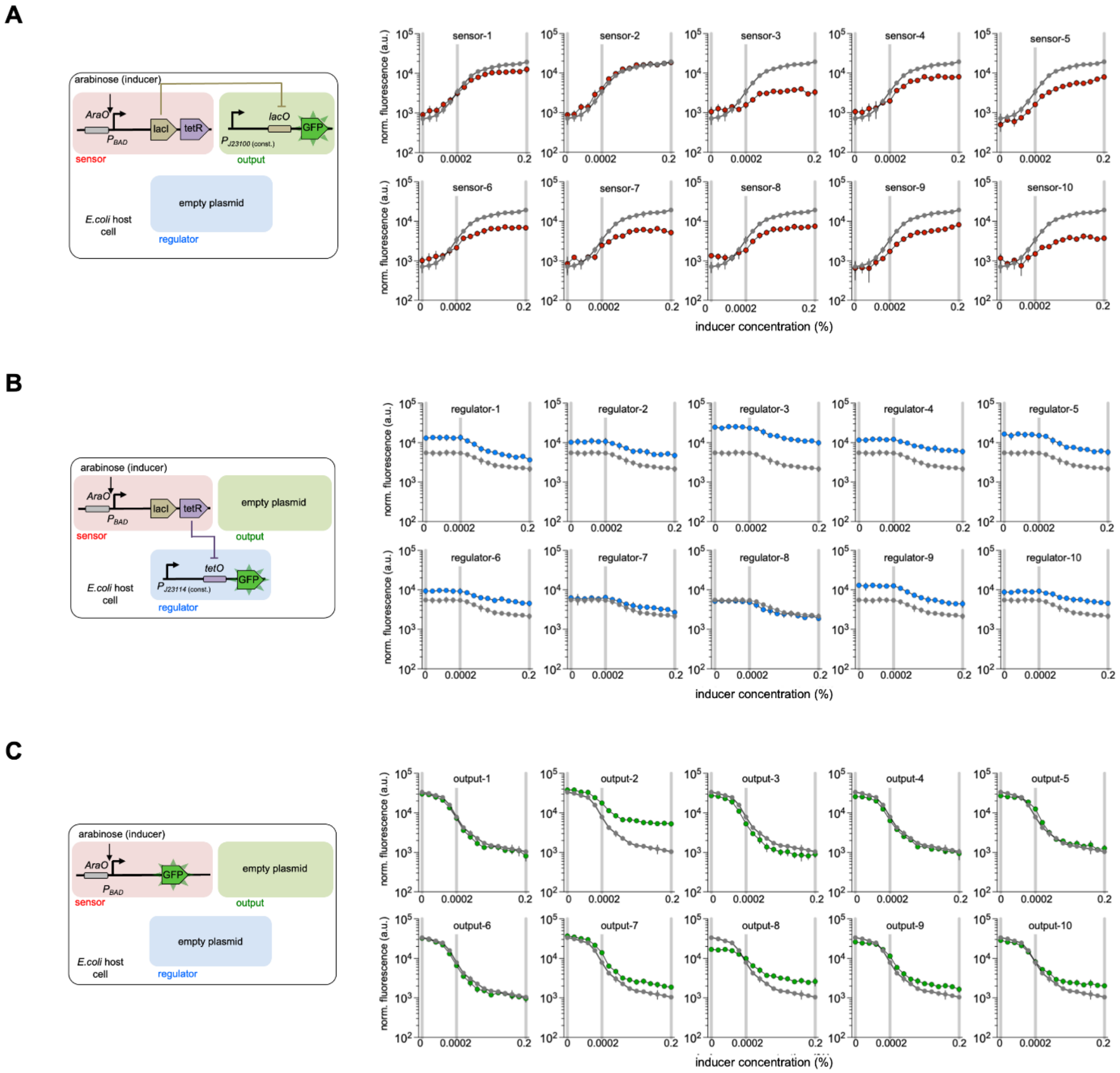

Fig. S3. Topology of partial networks to study the effect of the mutations in the promoter and operator regions on the expression levels. (**A**) To study the sensor genotypes we replaced *lacI* and *tetR* with *gfp*. The sensor is the only functional regulatory region present. Right: GFP expression. (**B**) To study the regulator genotypes we replaced *lacI* with *gfp* and kept the wild-type sensor node for inducer-dependent repression. Right: GFP expression. (**C**) To study the output genotypes we replaced the regulator plasmid with an empty pCDF plasmid and kept the wild-type sensor node for inducer-dependent repression. Right: GFP expression. Each genotype was measured in triplicate at 16 inducer concentrations and the mean and s.d. from three biological replicates are shown. The wild-type variant with no mutation in the regulatory region is shown in grey. In all cases we used the three plasmids with the corresponding antibiotic resistances.

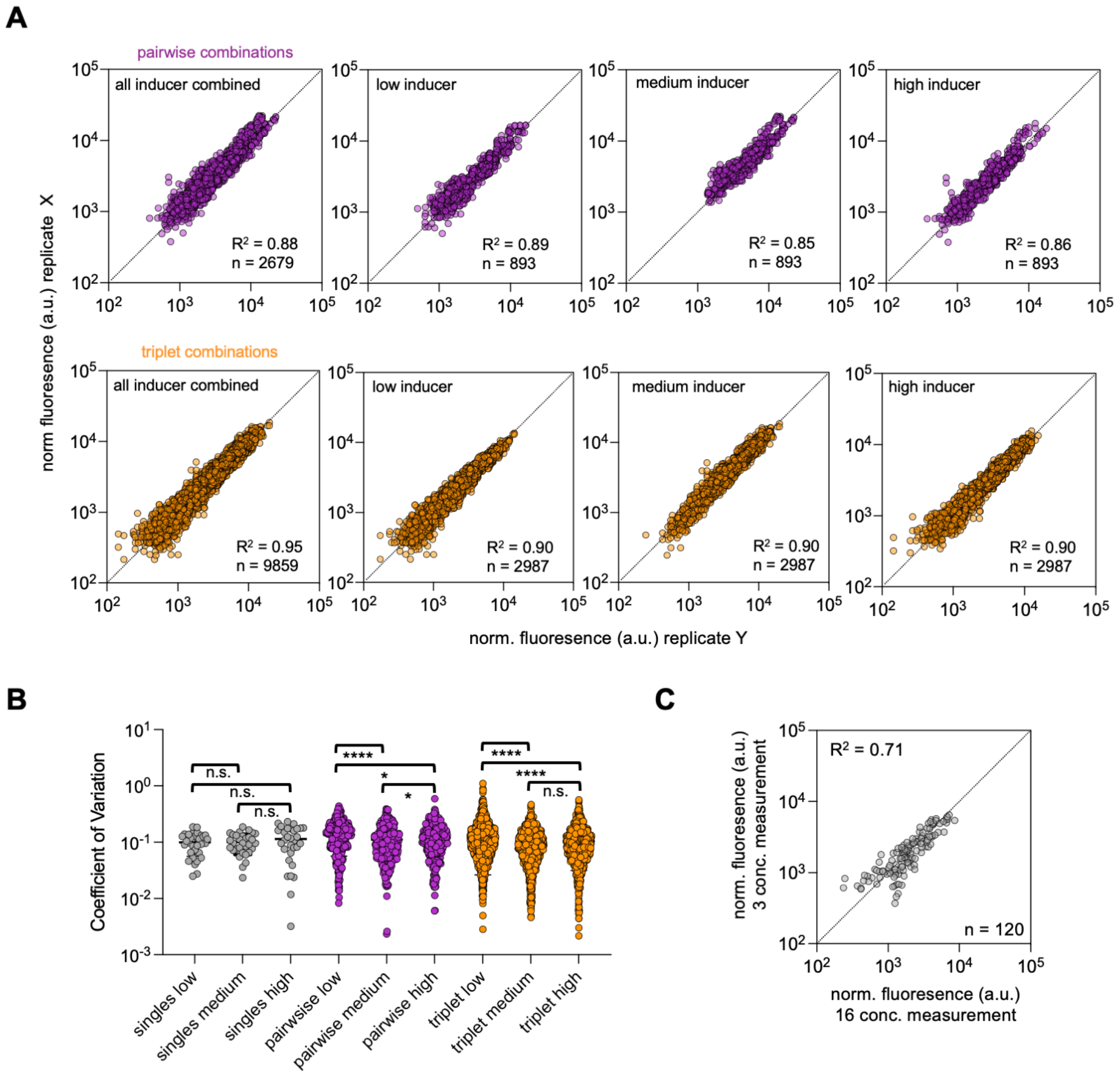

Fig. S4. Experimental reproducibility and variation of measurements. (**A**) Correlation between replicate measurements for all pairwise and triplet genotypes at different inducer concentrations and combined (left). Measurements were performed in triplicate and correlation shows comparison between replicate 1 and 2, 2 and 3, and 1 and 3. X means replicate 1 or 2. Y means replicate 2 or 3. (**B**) Comparison of coefficient of variation (standard deviation divided by the mean) of fluorescence measurements between pairwise and triplet combinations for different inducer concentrations. Significance between inducer levels was calculated using unpaired t-test assuming non-Gaussian distribution (Mann-Whitney U test). N.s. means not significant p-value >0.05, * indicates p-values <0.05, ** indicates p-values <0.01, *** indicates p-values <0.001, and **** indicate p-values <0.0001. (**C**) Correlation between the two independent measurements at 3 and 16 inducer concentrations of 40 selected genotypes as shown in fig. S5.

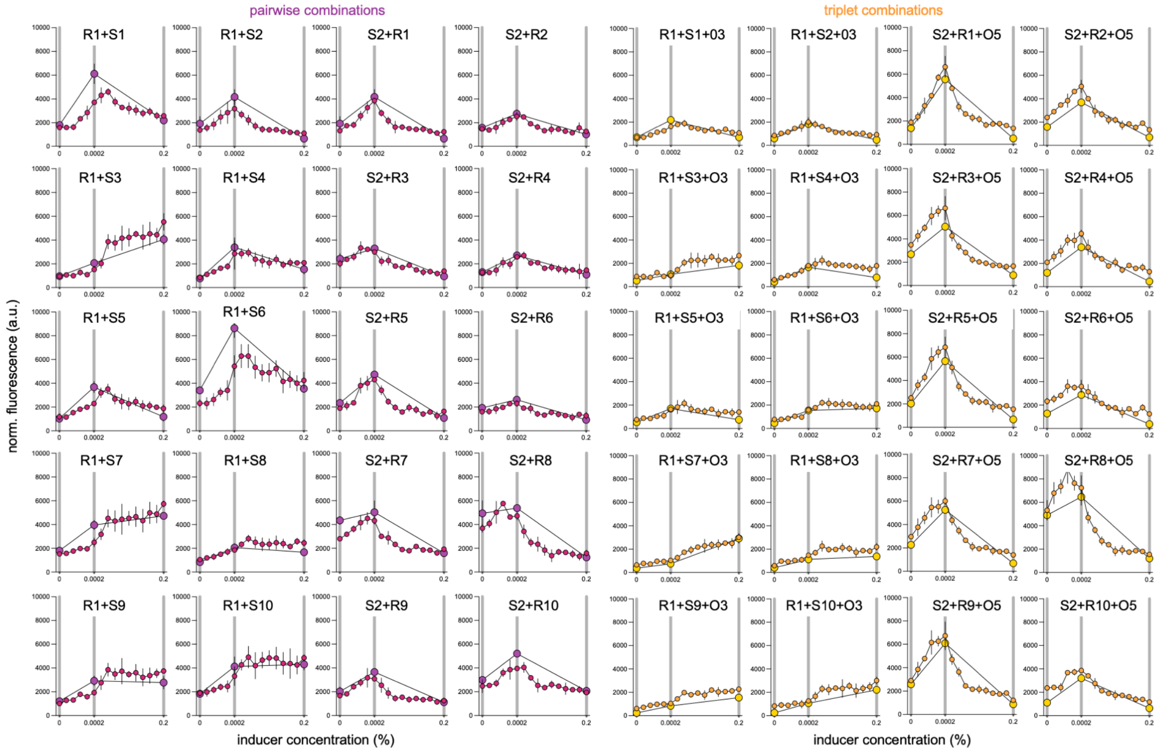

Fig. S5. Measured GFP expression pattern of 40 selected pairwise and triple genotypes at 3 and 16 inducer concentrations. We selected genotypes composed of combinations of regulator-1 with sensor-1 to sensor-10 (pairwise) and output 3 (triple), as well as genotypes composed of combinations of sensor-2 with regulator-1 to regulator-10 (pairwise) and output 5 (triple). We chose these genotypes to cover a wide diversity of pattern phenotypes. Each genotype was measured in triplicate at 3 (large points) and 16 (small points) inducer concentrations and the mean and standard deviation from three biological replicates are shown. The correlation of the measurements at low, medium and high inducer concentrations is shown fig. S4C.

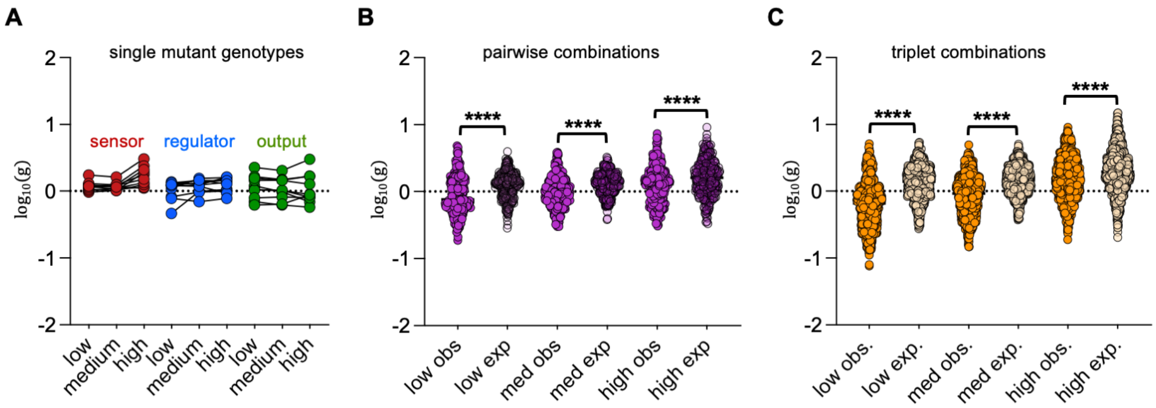

Fig. S6. Relative change in fluorescence levels for all genotypes and combinations at different inducer concentrations. (**A**) Relative change in fluorescence (log10 (g)) of the 30 single mutant genotypes at low (0%), medium (0.0002%) and high (0.2%) inducer concentrations. Lines connect the same genotypes at different inducer concentrations. (**B**) Relative change in fluorescence (log10 (g)) of observed (obs.) and expected (exp.) pairwise combinations. (**C**) Relative change in fluorescence (log10 (g)) of observed (obs.) and expected (exp.) triplet combinations. Asterisks indicate significant difference of variability between the observed and expected relative fluorescence values (significance calculated using an unpaired t-test assuming non-Gaussian distribution (Mann-Whitney U test)). N.s. means not significant p-value >0.05, * indicates p-values <0.05, ** indicates p-values <0.01, *** indicates p-values <0.001, and **** indicate p-values <0.0001.

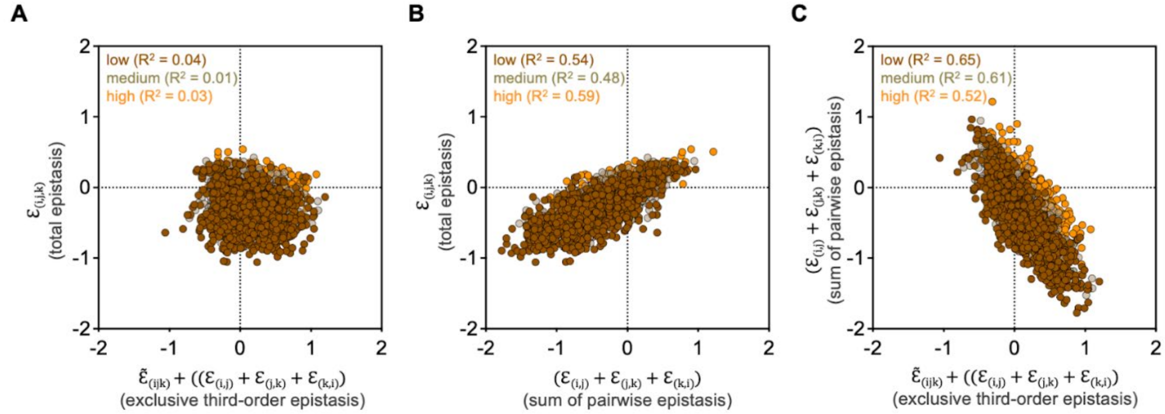

Fig. S7. Correlation of epistasis calculated for the triplet combination genotypes. (**A**) Total epistasis of triplet combinations (calculated from the single mutants) and the exclusive triplet epistasis. (**B)** Total epistasis of triplet combinations (calculated from the single mutants) versus the sum of pairwise epistatic effects. (**C**) Sum pairwise epistatic effects versus the exclusive third-order epistasis. Correlations (R^2) were calculated with Prism (Version 9.4.0, GraphPad Software, LLC.) using linear regression.

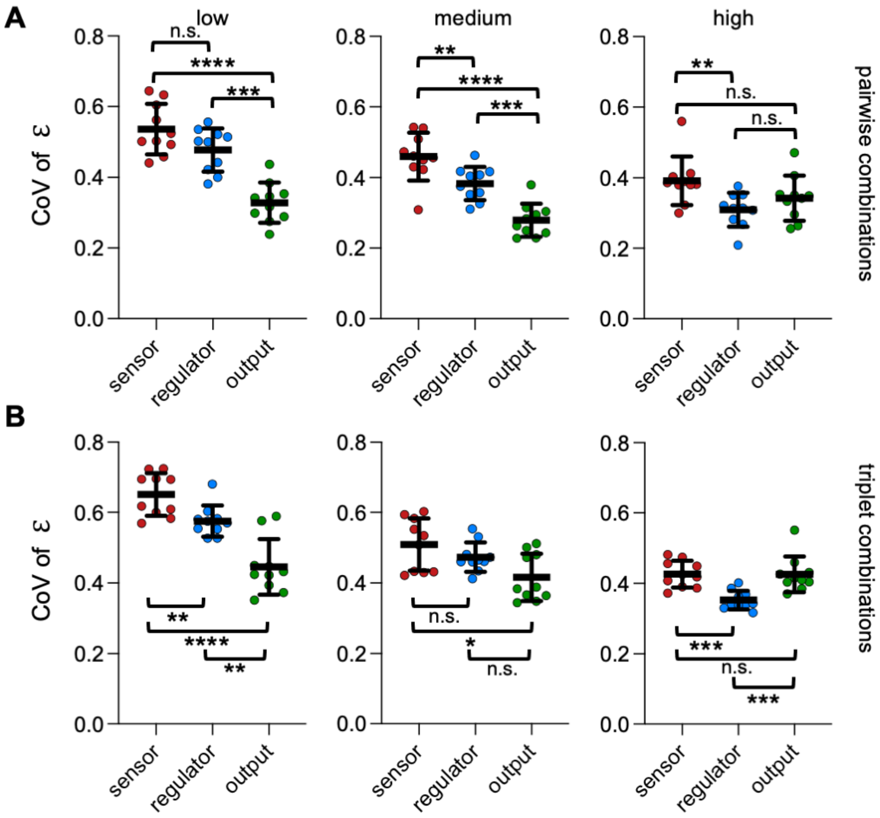

Fig. S8. Statistical analysis for coefficients of variation (CoV) of epistasis between different inducer concentrations. Shown separately for (**A**) pairwise combinations and (**B**) triplet combinations for the different regulatory nodes. Asterisk indicates significant difference of mean between the observed and expected relative fluorescence values (significance calculated using unpaired t-test assuming non-Gaussian distribution (Mann-Whitney test)). N.s. means not significant p-value >0.05, * indicates p-values <0.05, ** indicates p-values <0.01, *** indicates p-values <0.001, and **** indicate p-values <0.0001.

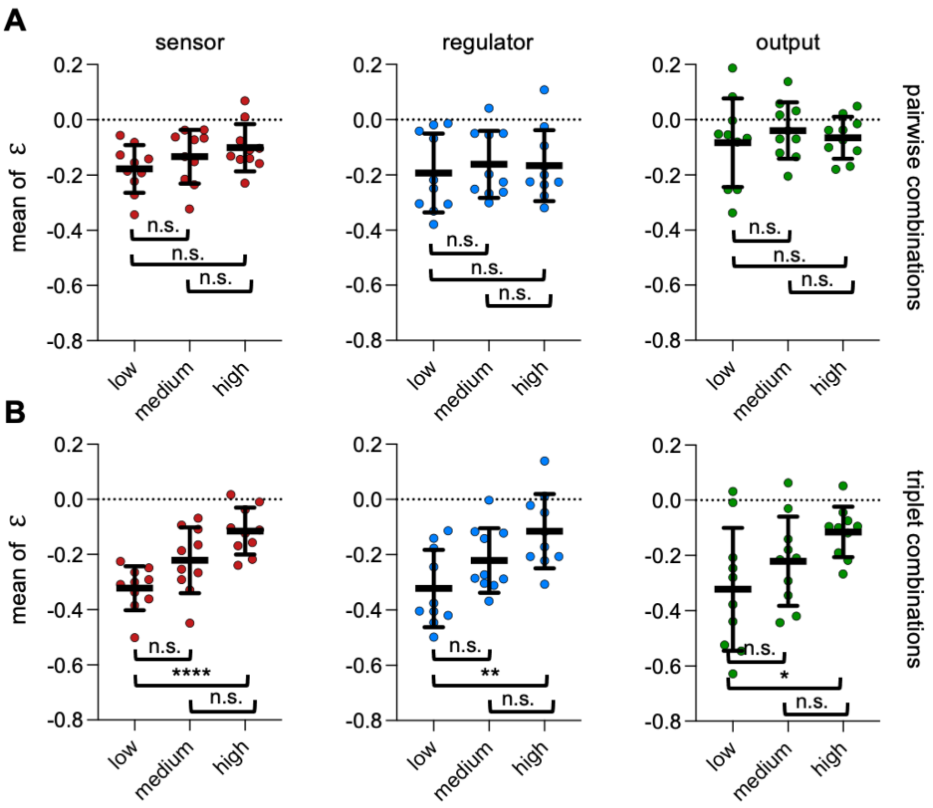

Fig. S9. Statistical analysis for mean values of epistasis between different inducer concentrations. Shown for (**A**) pairwise combinations and (**B**) triplet combinations for the different regulatory nodes. Asterisk indicates significant difference of mean between the observed and expected relative fluorescence values (significance calculated using unpaired t-test assuming non-Gaussian distribution (Mann-Whitney U test)). N.s. means not significant p-value >0.05, * indicates p-values <0.05, ** indicates p-values <0.01, *** indicates p-values <0.001, and **** indicate p-values <0.0001.

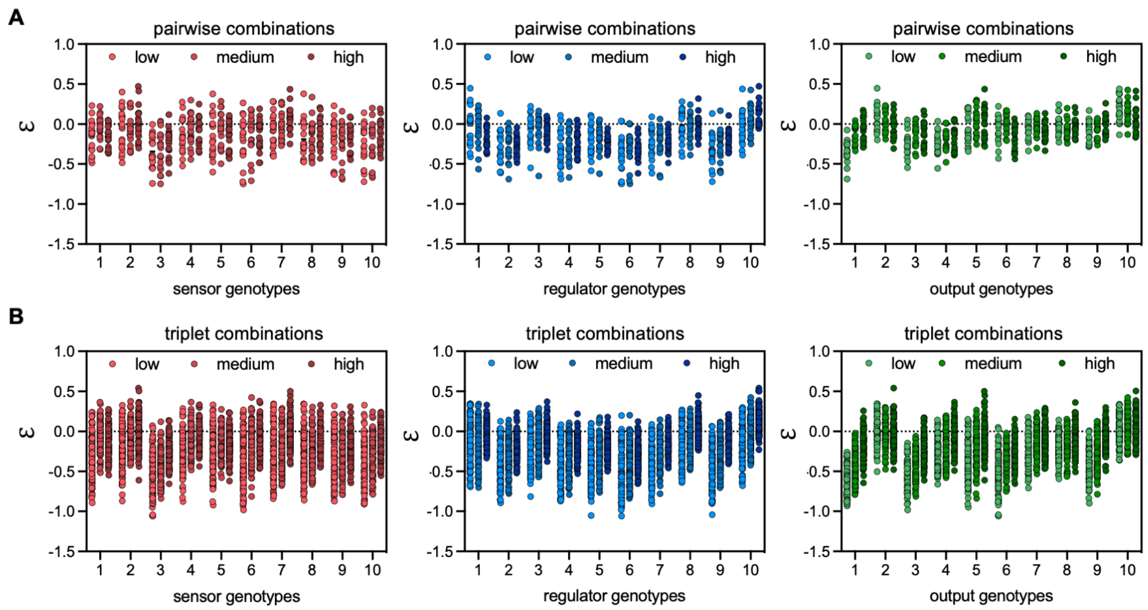

Fig. S10. Epistasis is inducer-dependent. Epistasis values for each mutant genotype and inducer concentration for (**A**) pairwise combinations and (**B**) triplet combinations.

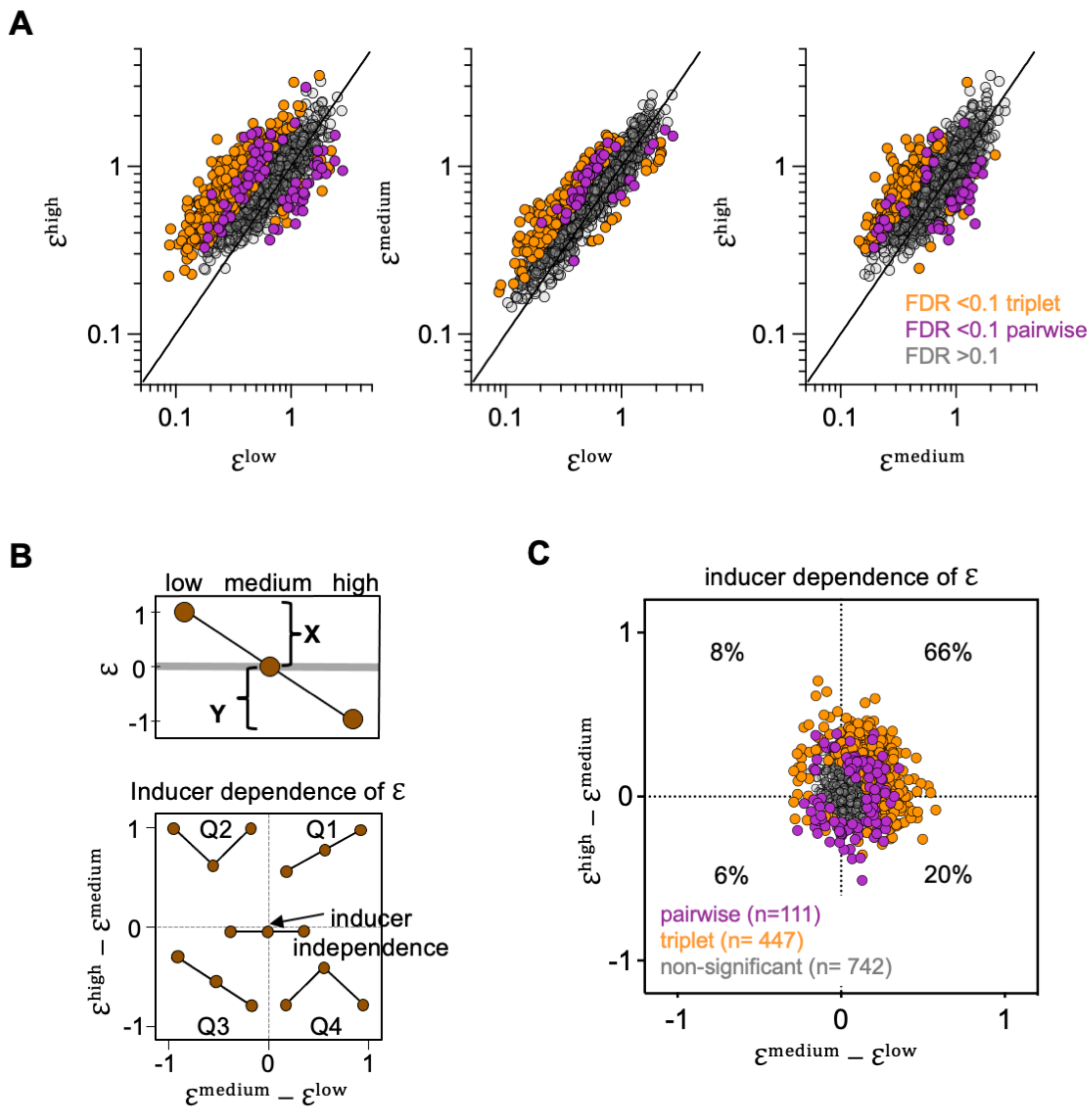

Fig. S11. Defining significant inducer-dependent epistasis. (**A**) Defining significant inducer-dependent epistasis of all 300 pairwise (purple) and 1000 triplet (orange) genotype combinations. Significance was calculated based on triplicate measurements with a series of t-tests (Welch's test) with FDR correction (n = 3900, Benjamini-Krieger-Yekutieli method) with significant values (FDR q-value <0.1) in colour and non-significant values (FDR q-value >0.1) in grey. At this significance cut-off, 37% of pairwise (111 of 300) and 45% of triplet (447 of 1000) combinations exhibited significant inducer-dependent epistasis. (**B**) Projection of inducer-dependence of epistasis (Ɛ) to two dimensional coordinates using ratios of Ɛ between medium-low (X axis) and high-medium (Y axis) inducer concentrations. (**C**) Genotypes with significant inducer-dependence are shown in color (pairwise in purple and triplet combinations in orange) and genotypes with non-significant inducer dependence are shown in grey.

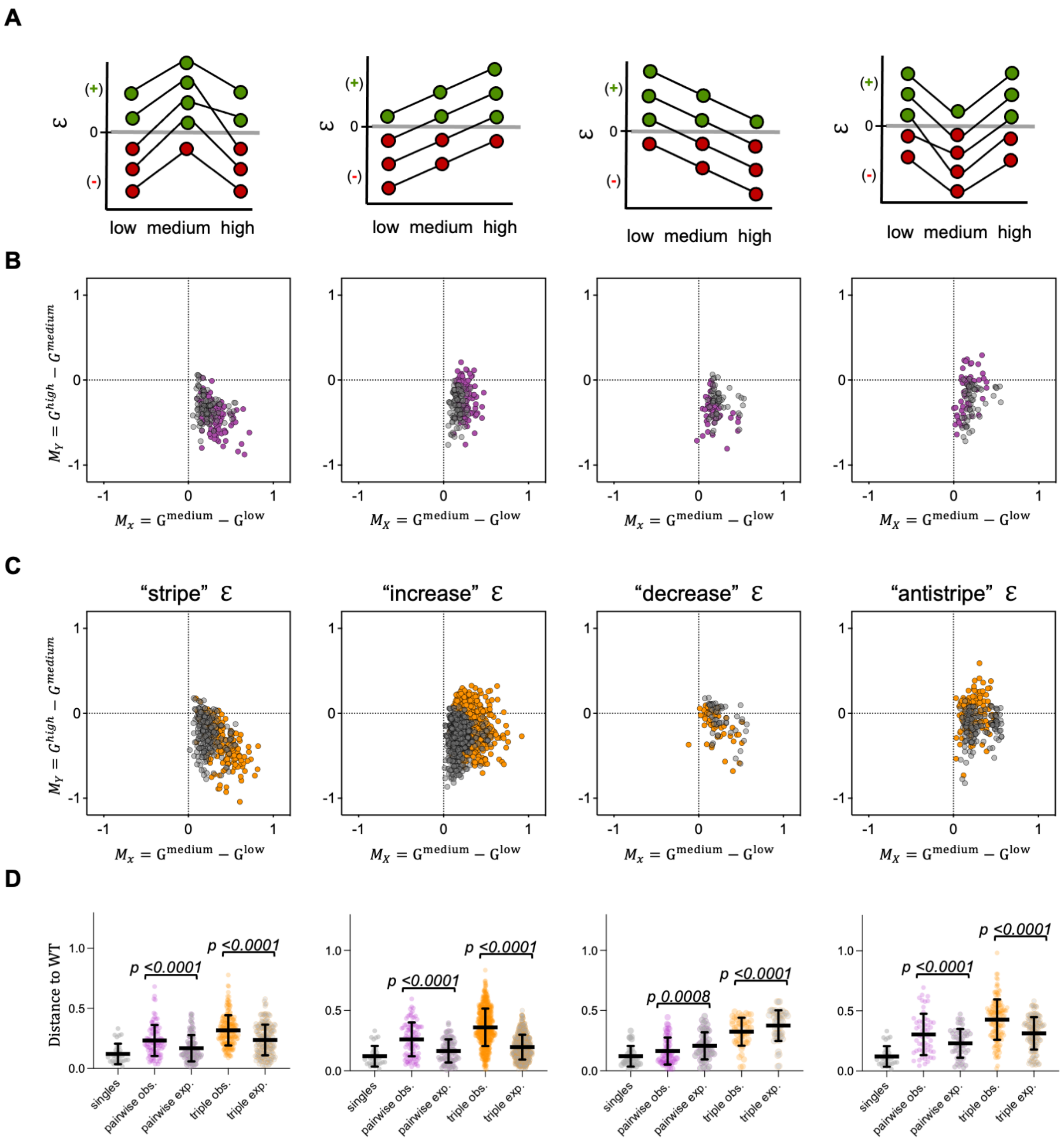

Fig. S12. Epistasis between inducer concentrations affects pattern phenotypes. (**A**) The different scenarios of epistasis values changing or maintaining sign across inducer concentration. (**B & C**) Projection of pattern phenotypes to two-dimensional coordinates using ratios of GFP expression between medium-low (X axis) and high-medium (Y axis) inducer concentrations. (**B)** Pattern phenotypes of pairwise combinations, separated by the different scenarios of epistasis across inducer concentrations. (**C**) Pattern phenotypes of triplet combinations, separated by the different scenarios of epistasis across inducer concentrations. (**D**) Measure of distance of pattern phenotypes relative to the wild type. Statistical significance (p-values) was calculated using a two-tailed paired t-test (Wilcoxon test).

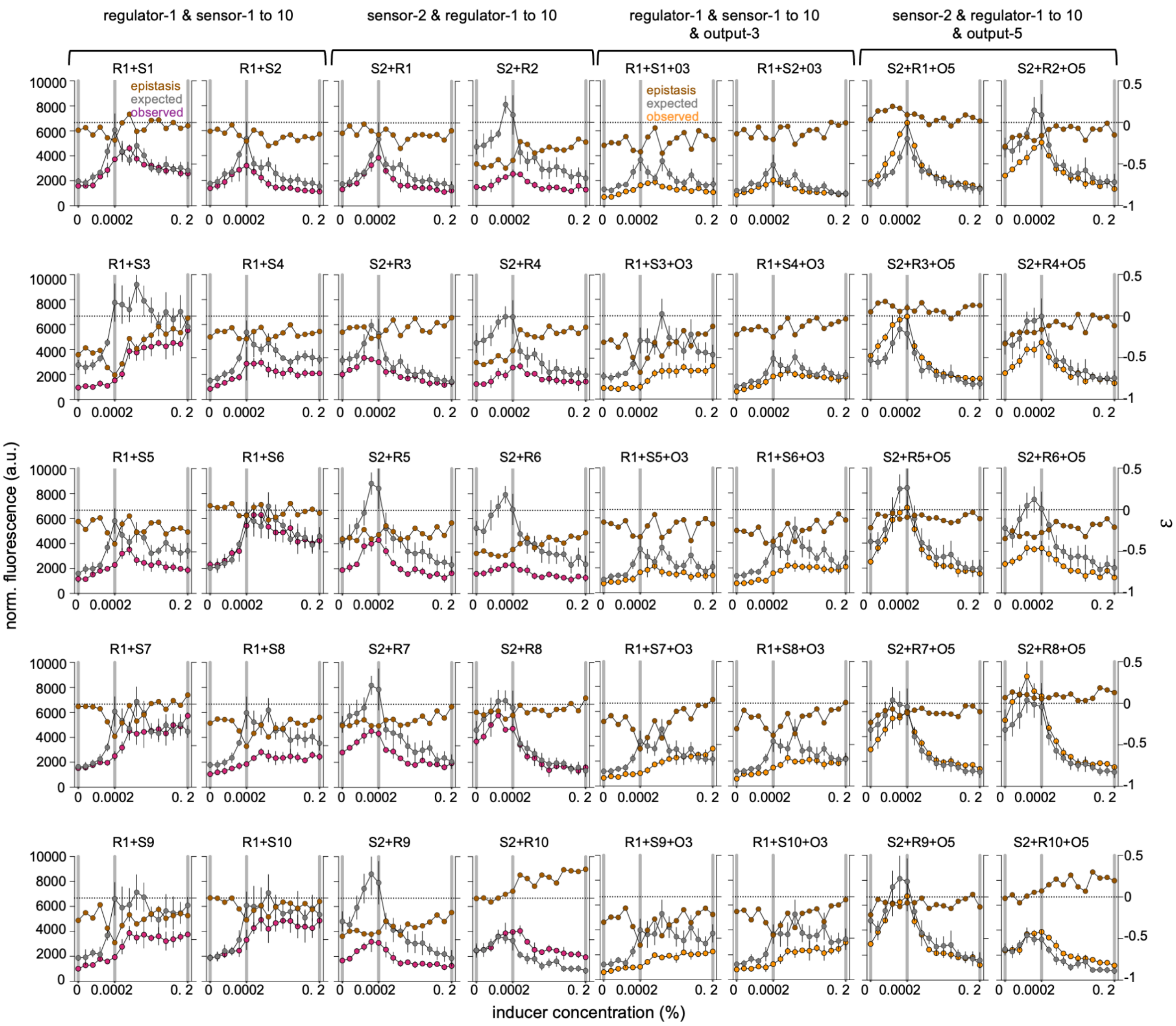

Fig. S13. Pattern phenotypes and epistasis along the inducer gradient for the selected combinations. The normalized fluorescence of the observed (pairwise in purple and triple in orange) and expected (grey nodes) mutant combinations are shown on the left y-axis. Their deviation is plotted as the resulting epistasis, shown in brown and scaled on the right y-axis. We find that most genotype combinations with regulator-1 have a clear dip of negative epistasis at medium inducer concentration which generally is the position of the highest GFP expression for a "stripe" phenotype. This strong epistasis results in changing the pattern phenotypes from a "stripe" to an "increase" pattern. For example, regulator-1 combined with sensor-3, switches from an expected "stripe" to an "increase" phenotype. The cause of this negative epistasis at low and medium inducer concentrations can likely be attributed to an overall higher LacI expression caused by a synergistic effect of both genotypes. First, regulator-1 has a lower sensitivity to TetR through a mutation in TetO, which results in higher LacI expression and consequently higher repression of the output node at low and medium inducer levels (fig. S5). In addition, sensor-3 has a lower sensitivity towards the inducer through two mutations in the AraC binding region, which results in lower TetR and LacI expression at medium and higher inducer levels and reduces the repression on the regulator node. This in turn increases and shifts the repression of

the output node by the regulator node towards higher inducer levels and changes the “stripe” to an “increase” pattern phenotype with much lower expression levels.

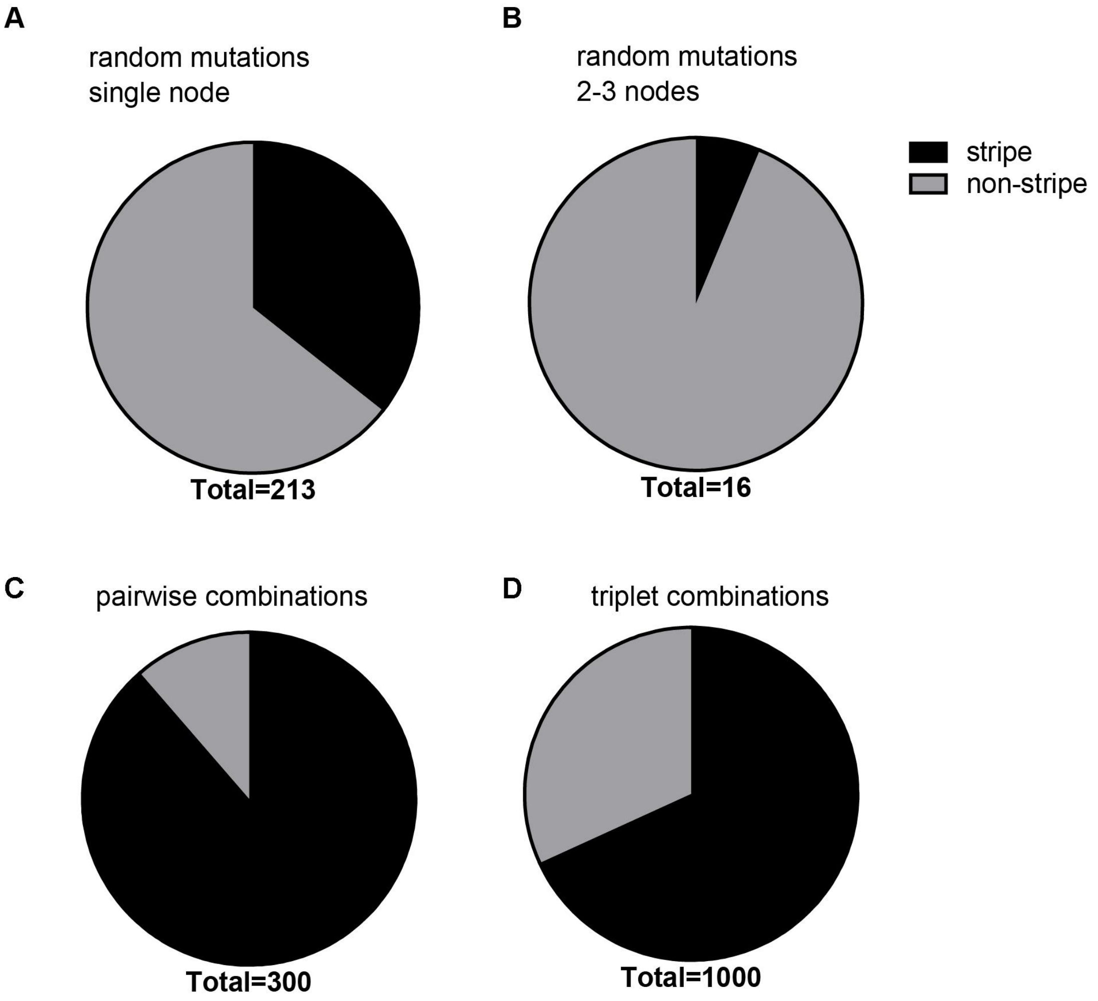

Fig. S14. Random mutations versus recombination. (**A**) Fraction of genotypes displaying a stripe or other phenotypes after introducing random mutations into a single node. Average number of mutations: 3.4. (**B**) Fraction of genotypes displaying a stripe or other phenotypes after introducing random mutations into 2-3 nodes. Average number of mutations: 6.0. (**C**) Fraction of genotypes displaying a stripe or other phenotypes after pairwise combinations in this study. Average number of mutations: 2.8 (**D**) Fractions of genotypes displaying a stripe or other phenotypes after triplet combinations in this study. Average number of mutations: 4.3. Data for (A,B) taken from Schaerli et al. (ref 39). Total: number of genotypes analyzed in each panel.

Source_Data. (separate file)
Source data for Figs. 1 to 5.
